# Supplementary material for: Content-rich biological network constructed by mining PubMed abstracts
Source: BMC Bioinformatics. 2004 Oct 8;5:147. doi: 10.1186/1471-2105-5-147 (PMC528731; doi:10.1186/1471-2105-5-147)
Supplement: Additional File 2 — The original results of the above study (non-essential files are deleted to keep the file size under the limit set by BMC bioinformatics). [file 1471-2105-5-147-S2.bz2 › chilibotAdditionalFile2/dip10/587ID9546397E10956/html/HFE_B2M.html]

 


 **HFE** and **B2M** 
  
Found 31 abstracts in PubMed, retrieved 10.  
 

 What does Google say? 
 PDF only 
| .edu only 

---

**Interactive relationship** (e.g. stimulation, inhibition, etc)

**Neutral relationship**- **HFE**  is a nonclassical class I molecule that associates with beta 2 microglobulin  [ **B2M** ]  beta 2m and with the transferrin receptor.  Ref: 12456502 Blood, 2003
- Most patients are homozygous for a C282Y point mutation that abrogates the interaction of  **HFE**  with beta 2 microglobulin  [ **B2M** ]  beta 2 M and, thus, impairs its proper processing and expression on the cell surface.  Ref: 12464008 Biochem J, 2003
- The presence of beta 2 microglobulin  [ **B2M** ]  at the apical plasma membrane corresponds to the recently observed association of beta 2 microglobulin  [ **B2M** ]  with  **HFE**  and FcRn.  Ref: 11935295 Histochem Cell Biol, 2002

**Non-interactive relationship** (e.g. studied together, co-existance, homology, etc.)

- The non covalent association of beta 2 microglobulin  [ **B2M** ]  with MHC class I molecules and MHC class I type molecules such as FcRn or the hemochromatosis  [ **HFE** ]  protein  **HFE**  is of major importance for their function, i.e., antigen presentation, IgG transport, and regulation of iron uptake, respectively.  Ref: 11935295 Histochem Cell Biol, 2002
- The function of  **HFE**  protein is unknown, but it is hypothesized that it acts in association with beta 2 microglobulin  [ **B2M** ]  and transferrin receptor 1 to regulate iron uptake from plasma transferrin by the duodenum, the proposed mechanism by which body iron levels are sensed.  Ref: 11943867 Proc Natl Acad Sci U S A, 2002
- To determine its role in hereditary hemochromatosis  [ **HFE** ]  HH, we used beta2 microglobulin knockout  **B2M**  mice that accumulate iron as in HH.  Ref: 12181184 Am J Physiol Gastrointest Liver Physiol, 2002
- The haemochromatosis  [ **HFE** ]  protein  **HFE**  induces an apparent iron deficient phenotype in H1299 cells that is not corrected by co expression of beta 2 microglobulin  [ **B2M** ] .  Ref: 12464008 Biochem J, 2003
- The following candidate genes have been examined in detail to determine whether polymorphisms in them may be responsible for this variation transferrin, transferrin receptor 1, transferrin receptor 2, ferritin L, ferritin H, IRP1, IRP2,  **HFE** , beta 2 microglobulin  [ **B2M** ] , mobilferrin calreticulin, ceruloplasmin, ferroportin, NRAMP1, NRAMP2 DMT1, haptoglobin, heme oxygenase 1, heme oxygenase 2, hepcidin, USF2, ZIRTL, duodenal cytochrome b ferric reductase DCYTB, TNFalpha, keratin 8, and keratin 18.  Ref: 12547238 Blood Cells Mol Dis< MedlineTA>Blood Cells Mol Dis, 2003
- Functional data suggest that the mutation C282Y abolishes the association of the  **HFE**  protein with beta 2 microglobulin  [ **B2M** ]  beta 2M, making the complex unable to reach the cell surface.  Ref: 11941597 Zhonghua Yi Xue Yi Chuan Xue Za Zhi, 2002
- We have moreover carried out a molecular analysis of candidate genes beta 2 microglobulin  [ **B2M** ] ,  **HFE** , and haem oxygenases 1 and 2 implicated in iron metabolism.  Ref: 11546828 J Med Genet, 2001
